# Crystal Structure and Optical Properties of ZnO:Ce Nano Film

**DOI:** 10.3390/molecules27165308

**Published:** 2022-08-20

**Authors:** Mei Xin

**Affiliations:** 1 School of Physics and Materials Engineering, Dalian Nationalities University, Dalian 116600, China; xinmei_dl@126.com; 2 Liaoning Key Laboratory of Optoelectronic Films and Materials, Dalian Nationalities University, Dalian 116600, China

**Keywords:** ZnO:Ce, thin film, sol-gel, nanocrystal, optical properties, blue emission, color purity

## Abstract

ZnO and cerium-doped ZnO on a glass substrate have been prepared by the sol–gel method using the spin coating technique and water bath growth process. Ce-doping concentration on film structure, morphology, and optical properties is investigated. The result indicated that the hexagonal wurtzite ZnO with high crystalline quality formed on the substrate. The crystal parameters a and c decreased, crystal size increased, and the compressive strain formed after Ce-doping. Formed un-, 3%, 6%, 12% Ce-doped ZnO film has a spherical shape with a size between 8.6–31, 14–52, 18–56, and 20–91 nm, respectively. All films had good absorption of 300–400 nm ultraviolet light, in particular, the absorption of near ultraviolet (370–400 nm) increased after doping of Ce. The transmittance of light between 400–800 nm decreased with Ce-doping concentration. The band gap energy increased after Ce-doping reaching better optical behavior for preparing ZnO heterostructured thin-film. All film emitted intense blue emission under 375 nm excitation at room temperature. This indicated the film can have application in optoelectronic devices.

## 1. Introduction

Zinc oxide (ZnO) has a band-gap width of 3.37 eV at room temperature (RT) and an exciton energy of 60 meV. ZnO has excellent chemical stability because of its hexagonal wurtzite structure at normal temperatures leading to a large Coulomb force for its positive and negative electron pairs [1]. ZnO is environmentally friendly, inexpensive, and abundant in nature and intrinsic defects such as oxygen vacancies or Zn interstitials are mostly on the surface of ZnO causing visible emissions [2,3]. All these unique properties make ZnO application in the ultraviolet (UV) and blue source and have efficient exciton emission [4]. ZnO has also attracted research interest for its applications in optoelectronic applications by forming homo, pn junction, and heterogeneous materials [5,6,7], and gas detection [8,9]. ZnO is generally an n-type semiconductor that usually forms a heterojunction LED with GaN, SiC, and other p-type semiconductors, and is used in optoelectronic devices [10]. For some of their specific application requirements, ZnO film requires specific structural features, morphology, and optical band gaps [11]. Doping is a significant and effective way to improve the physical properties of ZnO thin films [12]. Cerium-doped ZnO is widely used due to its unique properties including visible-light-emitting devices [13,14], catalytic characteristic [15,16,17], new diluted magnetic [18], spintronics [19,20] and gas sensor [21]. Various synthesis techniques have been used to prepare ZnO nanoparticles, such spray pyrolysis [22], RF magnetron sputtering [23], solid-state reaction [24], solution method [25,26], electrospinning [27] co-precipitation [28], sol–gel [29,30]. In this study, we report the Ce concentration on structural, morphological, and optical properties of sample films deposited by the sol–gel method combining the spin coating technique and water bath growth. This process can reduce spin coating time, simplicity, and uniformity film on glass substrates. Few reports about affect Ce concentration on the crystal structure of ZnO:Ce film. In this work, the effect of Ce-doping on the crystal structure and the optical properties of ZnO:Ce were studied in detail.

## 2. Experimental Process

### 2.1. The Deposition of Un- and Ce-Doped ZnO Film on Glass Substrates

**Figure 1:**
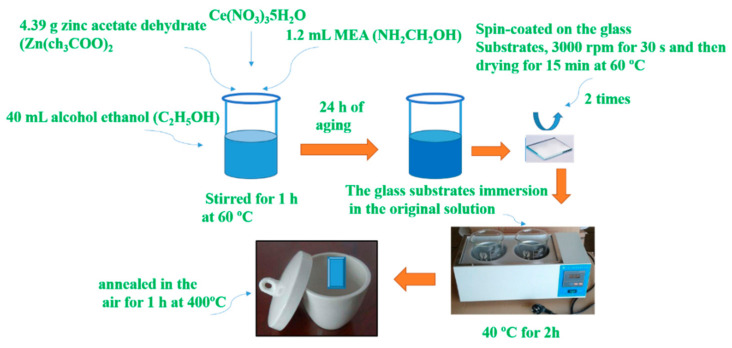
Schematic diagram for the deposition of un- and Ce-doped ZnO film on glass substrates.

All reactants were reagent grade (AR). Ce-doped sample added 3%, 6%, 12% of Ce(NO_3_)_3_5H_2_O to solution. The deposition processes as Figure 1.

### 2.2. Film Characterization

The structure and morphology were examined by using an XRD (SHIMADZU-6000) and SEM (Hitachi S4800). UV/vis transmittance and absorption of the sample were performed using UV-3600 SHIMADZU spectrophotometer. PL spectra were measured by HITACHI F-4600 spectrophotometer. All measurements were performed at room temperature.

## 3. Results and Discussion

### 3.1. XRD and SEM Analysis

**Figure 2:**
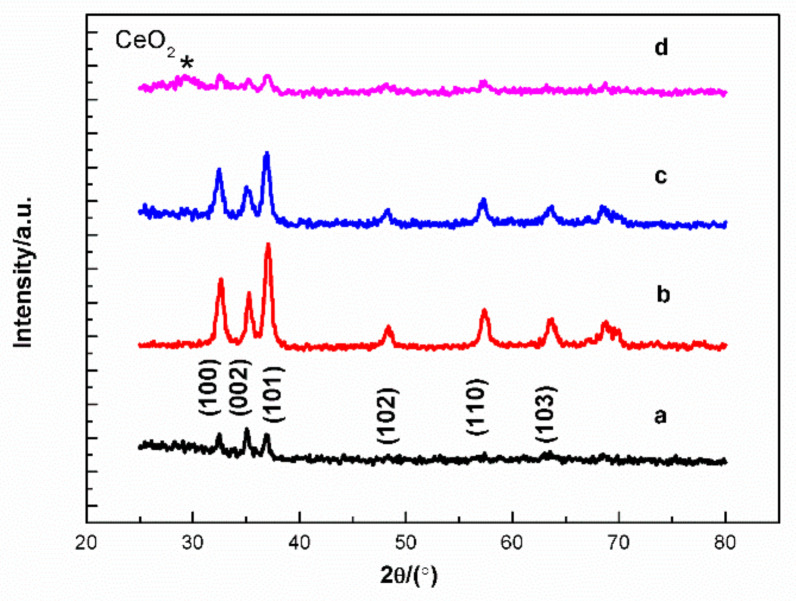
XRD patterns of un-(**a**), 3% (**b**), 6% (**c**) and 12% (**d**) Ce-doped ZnO film.

Figure 2 shows the XRD patterns of un-, 3%, 6% and 12% Ce-doped ZnO film. The films are well matched with hexagonal wurtzite ZnO (JCPDS 36-1451) [29]. The strongest diffraction peak is obtained at 3% Ce-doped sample and the diffraction peak intensity decreases with further increasing of Ce contents. When Ce-doping ratio is 12%, CeO_2_ impurity appeared (2θ–29°) marked as * in Figure 2 [16,29]. Ce-doped ZnO film oriented along (101) crystallographic plane. The lattice constants calculated using:

(1) $$\frac{1}{d_{hkl}^{2}}=\frac{4}{3}\left(\frac{h^{2}+hk+k^{2}}{a^{2}}\right)+\frac{l^{2}}{c^{2}}\;\;\;\;$$

The crystallite size (D) is calculated using the Debye–Scherrer’s formula for the average calculated date of the (100), (002) and (101) diffraction peaks [31].

(2) $$D=\frac{0.89\lambda }{\beta cos\theta }$$

(3) $$\varepsilon_{zz}=\frac{\left(c-c_{0}\right)}{c_{0}}\times 100\%$$

where *ε_zz_* is along the *c* axis, *c*_0_ and *c* is the lattice parameter of un- and Ce-doped ZnO film.

**Figure 3:**
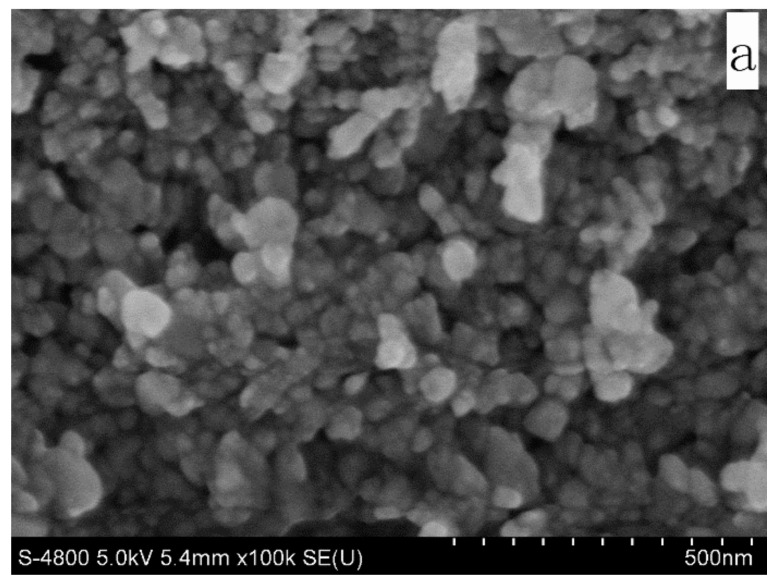

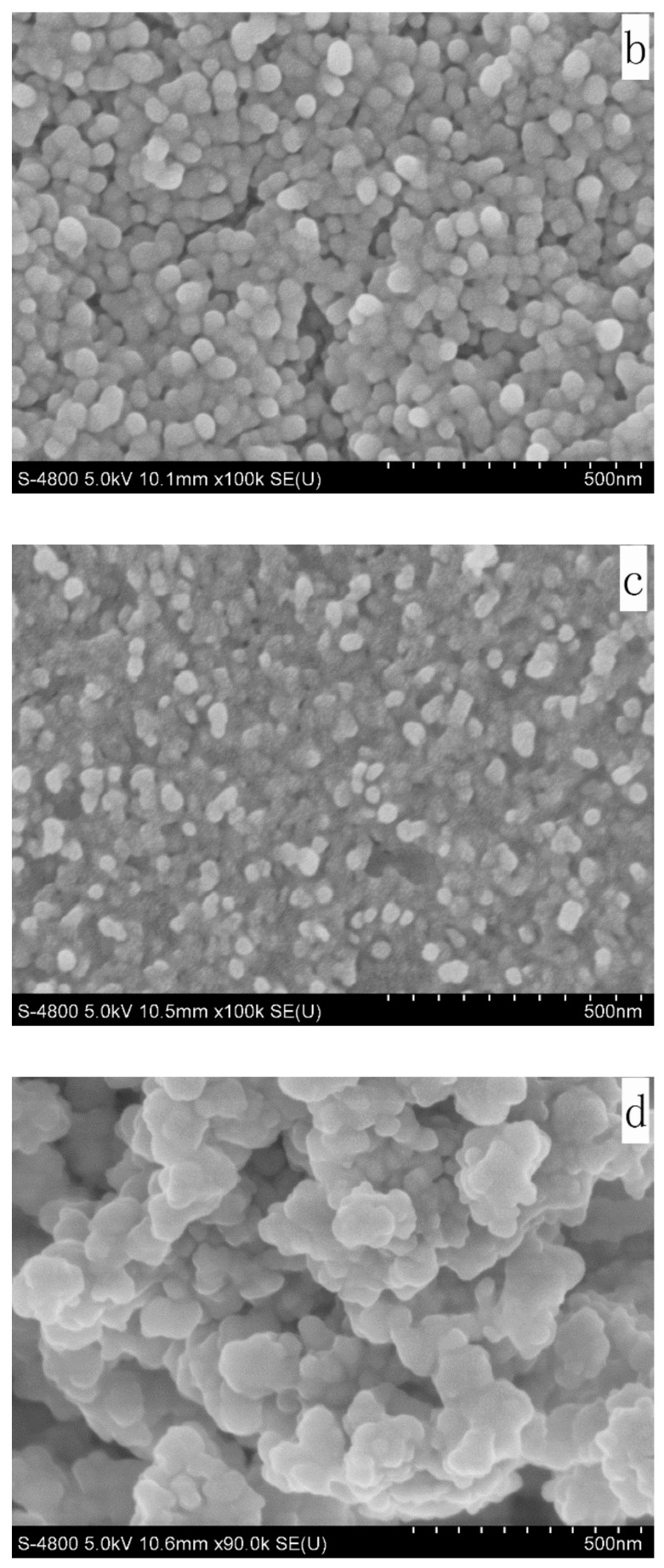
SEM of un-(**a**) 3% (**b**) 6% (**c**) 12% (**d**) Ce-doped (**b**) ZnO thin film.

The crystal size increased and lattice constant decreased after Ce doping (Table 1). The ionic radii of Ce^4+^ and Ce^3+^ (0.087 and 0.115 nm, respectively) be larger than that of the Zn^2+^ ion of 0.074 nm [1]. The compressive strain is formed after doping of Ce. Changes in crystal structure after doping Ce indicate that Ce is incorporated into the ZnO matrix. SEM result indicated that film morphology of un-, 3%, 6% and 12% Ce-doped ZnO film diameter range in 8.6–31, 14–52, 18–56 and 20–91 nm, respectively (Figure 3). A hundred particles were selected and measured by Image J software. The 3% and 6% Ce-doped samples appear with a spherical morphology. There is a local agglomeration in the undoped and 12% Ce-doped sample, and the uniformity of the film after doping 3% and 6% Ce is obtained. In our previous work, the morphology of doped ZnO was changed from granular to rod when doping concentration is larger than 1% deposited on glass substrate by similar method [32,33,34]. However, in this work, the sample morphology do not change after larger than 1% Ce-doping, as well as lattice constant decreased after doping of larger ionic radii of Ce indicating the unique properties of Ce ions. It is maybe due to the dominant compressive strain on ZnO lattice caused by Ce enclosed in grain boundaries and prevents the growth further along the growing crystal orientation [35,36].The particle size increased after doping of Ce, consistent with the calculated size trend.

### 3.2. Optical Properties

**Figure 4:**
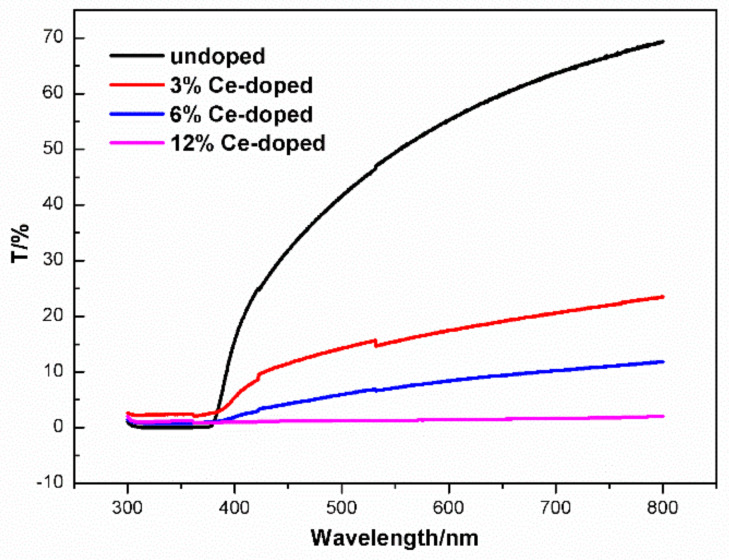
UV-vis optical transmittance of un-and Ce-doped ZnO films.

**Figure 5:**
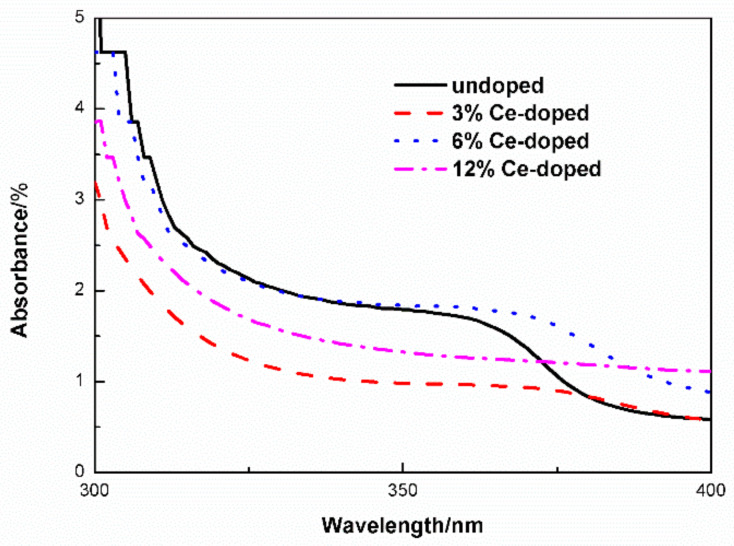
UV-vis absorbance of un-and Ce-doped ZnO films.

The film completely absorbs the ultraviolet (UV) light and transmits the visible light. The visible light transmittance decreases with increasing of Ce content (Figure 4). Numerous factors can influence film visible light transmittance. In our experiment, the decreased visible light transmittance is maybe due to crystal size increased after Ce-doping. UV-vis absorbance of un-and Ce-doped ZnO film displayed as Figure 5. The absorbance peak appears at about 300 nm. The absorption of near ultraviolet (370–400 nm) is stronger than the undoped one after doping of Ce ions. Optical gap values for un- and Ce-doped ZnO using the well-known Equation (4)

(4) $$\alpha \mathrm{hv}=A{\left(\mathrm{hv}-E_{g}\right)}^{1/2}$$

**Figure 6:**
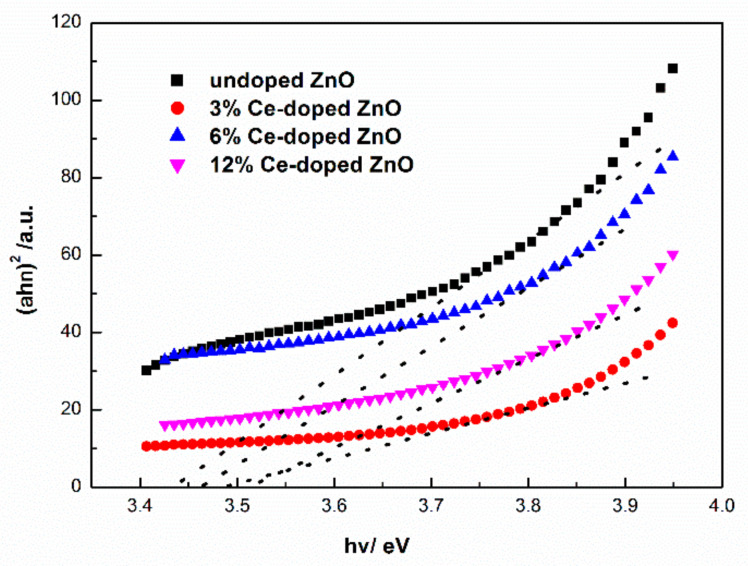
Optical band gap (a is intercepted between 314–376 nm) of un- and different Ce-doped ZnO films.

The value of absorption coefficient (a) is intercepted between 314–376 nm for calculating the band gap (*E_g_*). The band gap of undoped, doping of 3%, 6% and 12% Ce ZnO is found to be 3.43, 3.49, 3.46 and 3.51 eV, respectively. The band gap increases after doping of Ce (Figure 6). The possible reason may relate to strain. The compressive strain widened band gap [37]. In addition, because of the Burstein–Moss effect also make the band gap widened [38]. The increase in the optical band gap brings it closer to the band gap width of p-type semiconductor materials and reaching better optical behavior for preparing ZnO heterostructured thin-film [39].

**Figure 7:**
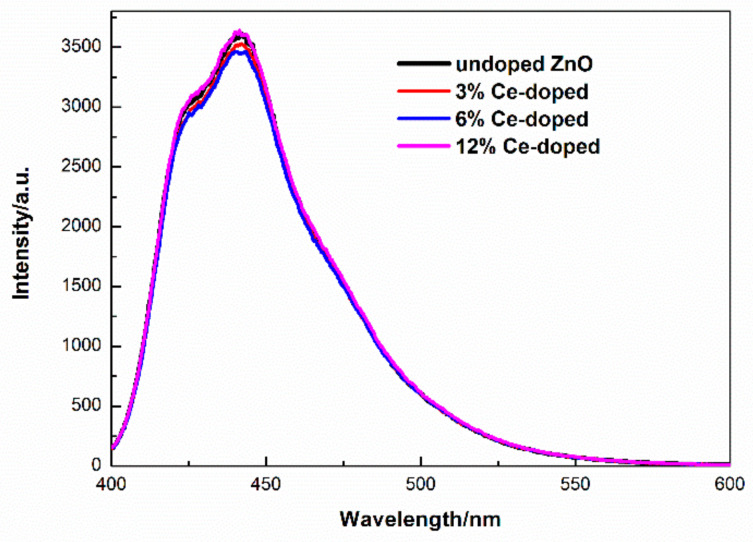
PL of un- and different Ce-doped ZnO films (λ_ex_ = 375 nm).

**Figure 8:**
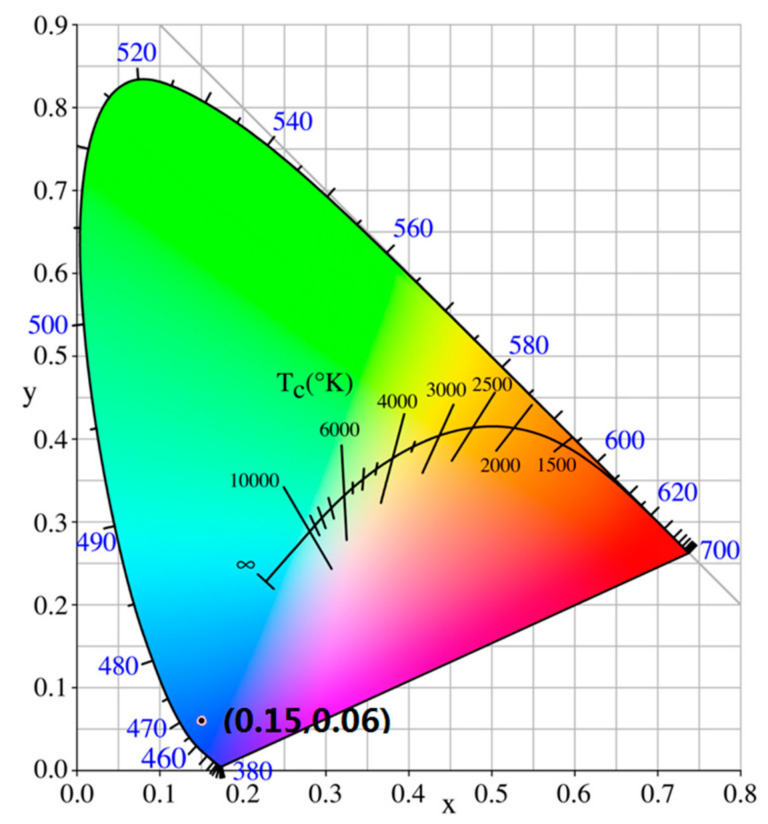
CIE-1931 chromaticity coordinates of samples excited under 375 nm.

The PL emission spectra of un-and different Ce-doped ZnO film under 375 nm excitation are shown in Figure 7. All films have emission peaks at about 424 and 442 nm. An emission of 424 nm is assigned to the recombination of the Zn interstitial levels to the top of the valence band; 442 nm emission is attributed to the recombination of O vacancies to the valence band [32]. CIE-1931 chromaticity coordinates of samples is (0.1518, 0.0594), (0.1517, 0.0602), (0.1518, 0.0603), (0.1519, 0.0598) for undoped and 3%, 6%, 12% Ce-doped ZnO, respectively (Figure 8). The color purity was calculated using [40]

(5) $$\mathrm{Color}\;\mathrm{Purity}=\frac{\sqrt{{\left(x_{s}-x_{i}\right)}^{2}+{\left(y_{s}-y_{i}\right)}^{2}}}{\sqrt{{\left(x_{d}-x_{i}\right)}^{2}+{\left(y_{d}-y_{i}\right)}^{2}}}\times 100\%$$

where (*x_s_*, *y_s_*) are the coordinates of a sample point; in our experiment all of the sample coordinates are basically the same, and the value is (0.152, 0.060), (*x_d_*, *y_d_*) are the coordinates of the dominant wavelength, in our experiment dominant wavelength at 442 nm, its coordinate is (0.1756, 0.0053) and (*x_i_*, *y_i_*) are the coordinates of the CIE coordinates of white illuminant point is (0.310, 0.316). The calculated colour purity is 89%, indicating that this material has potential application in blue light sources.

## 4. Conclusions

In summary, undoped, Ce-doped ZnO films deposited on glass substrates were synthesized by sol–gel method contained spin coating and water bath growth technique. The Ce incorporation on the crystal structure and optical properties of un- and Ce-doped ZnO film was investigated. XRD results indicated that all films formed a hexagonal wurtzite ZnO crystal structure, the lattice constant decreased, crystal size increased, and compressive strain formed after Ce-doping, indicating the Ce incorporated into ZnO crystal. The presence of spherical type was confirmed by SEM. Room temperature UV–vis and PL spectra showed strong absorption in the near UV region and the absorption of near ultraviolet (370–400 nm) increased after doping of Ce ions. Optical transmittance of visible light was reduced with increasing Ce-doping content. The band gap energy increased after Ce-doping. Intense blue emission with a color purity of 89% was observed under 375 nm excitation. ZnO:Ce film has potential applications in the near ultraviolet (n-UV) LED light conversion materials and a blue light source.

## Figures and Tables

**Table 1:** Lattice constant and diameter of undoped and Ce-doped ZnO thin film.

Molar Ratio of Ce (%)	Lattice Constant			*ε_zz_*	Debye–Scherrer’s D (nm)	SEM G (nm)
	*a*	*c*	*c/a*			
0	0.2995	0.4815	1.61	0	14.93	8.6–31
3	0.2972	0.4781	1.61	−0.70	17.94	14–52
6	0.2989	0.4806	1.61	−0.20	16.03	18–56
12	0.2989	0.4784	1.60	−0.50	23.79	20–91

## Data Availability

Not applicable.
